# Psychosocial Basis of Human Sufferings and Poverty in Patients with Neurological and Psychiatric Disorders

**DOI:** 10.18103/mra.v11i5.3919

**Published:** 2023-05-20

**Authors:** Souvik Dubey, Ritwik Ghosh, Mahua Jana Dubey, Shambaditya Das, Arka Prava Chakraborty, Arindam Santra, Ajitava Dutta, Dipayan Roy, Alak Pandit, Biman Kanti Roy, Gautam Das, Julián Benito-León

**Affiliations:** 1 Department of Neuromedicine, Bangur Institute of Neurosciences (BIN), Kolkata, West Bengal, India;; 2 Department of General Medicine, Burdwan Medical College, and Hospital, Burdwan, West Bengal, India;; 3 Department of Psychiatry, Berhampur Mental Hospital, Berhampur, West Bengal, India;; 4 Indian Institute of Technology (IIT) Madras, Chennai, Tamil Nadu, India;; 5 School of Humanities, Indira Gandhi National Open University (IGNOU), New Delhi, India;; 6 Department of Neurology, University Hospital “12 de Octubre”, Madrid, Spain;; 7 Research Institute (i+12), University Hospital “12 de Octubre”, Madrid, Spain;; 8 Centro de Investigación Biomédica en Red Sobre Enfermedades Neurodegenerativas (CIBERNED), Madrid, Spain;; 9 Department of Medicine, Complutense University, Madrid, Spain

**Keywords:** Poverty, Human Suffering, Neurological disorders, Psychiatric disorders

## Abstract

Neurological disorders and psychiatric ailments often lead to cognitive disabilities and low attainment of education, pivoting misconceptions, myths, and misbeliefs. Poverty and low educational attainment are intriguingly associated with poor awareness and perception of these diseases that add to the suffering. Poverty goes parallel with a low level of education and is intricately associated with neuropsychiatric ailments, which have the potential to spread transgenerationally. Robust education policies, proper government rules and regulations against the spread of disease-related myths and misconceptions, uplifting medical education in its true sense, voices against consanguinity, and programs to raise scientific perception about diseases can help to throw light at the end of this dark tunnel. In this article, the authors intend to 1) decipher the potential psychosocial basis of human suffering and poverty in patients with neurological and psychiatric disorders, and 2) discuss the apropos way-outs that would potentially mitigate suffering, and alleviate the economic burden and cognitive disabilities of families with neuropsychiatric diseases.

## Introduction

Neurological disorders and psychiatric ailments have strong genetic predispositions. [1,2] This, in turn, leads to an ever-increasing cumulative burden of neurological and psychiatric diseases in families and the community at large due to a dire lack of genetic counseling. [1,2] In developing countries where genetic counseling is outside the reach of the majority, it has been an everlasting issue.

This statement seems to be a meagre oversimplification of the actual state of affairs the authors intend to disclose in this article. Stroke, epilepsy, and neuroimmunological demyelinating, neurometabolic, and degenerative diseases have specific neuroanatomical involvement and a pathological and molecular basis. [3–7] Interestingly, similar areas of neuroanatomical involvement and shared pathological and molecular basis have been observed in various psychiatric ailments/symptoms, which strongly argue against neural cubism and explain the basis of several clinical entities with overlapping neurological and psychiatric symptoms. [7–13] The fascinating area to discuss is allelic heterogeneity, which explains that different mutations of the same genetic locus can lead to manifestation of different disease phenotypes/phenocopies. [7–13] The underlying mechanism is either quantitative or qualitative gene mutation with a consequent impact on its downstream signals. [7–13] Extrapolation of allelic heterogeneity and pleiotropy in the neuropsychiatric continuum provide the best explanation for a family with a history of genetic neurological diseases may have psychiatric ailments or vice versa, considering overlapping clinical features and shared anatomical, pathological, and molecular basis of various neuropsychiatric ailments. This leads to an increased cumulative burden of neuropsychiatric diseases in families and society with significant deleterious social and economic consequences by the following possible mechanisms underpinning. [7–13] In this article, the authors intend to 1) decipher the potential psychosocial basis of human suffering and poverty in patients with neurological and psychiatric disorders (figure), and 2) discuss the possible way-outs that would potentially mitigate suffering, and alleviate the economic burden and cognitive disabilities of families with neuropsychiatric diseases.

### Genetic and epigenetic basis of cognitive disabilities:

The basic difference between central nervous system (CNS) disorders and the diseases of other systems is that cognitive functions and behavior of individuals are frequently jeopardized in the former and remain relatively stable in the later. The learning and education of society depend on individuals’ cognitive abilities, which are frequently disrupted in neurological ailments. Moreover, building blocks of behavioral development of individuals also get disrupted since early childhood in patients with neurological diseases. This is because healthier parenting is often not possible in a family with neuropsychiatric ailments as parents usually suffer from widely variable phenotypes/phenocopies of genetically governed neuropsychiatric issues. [14–18] Examples are ample. In children with genetic epilepsies, though a family history of epilepsy is not found most of the time (especially in developing and underdeveloped countries), studies and our life experience have demonstrated significant psychopathology/personality disorders among parents with epilepsy. [19–22] Moreover, parental psychopathology and related burden in children with autism spectrum disorder and attention-deficit/hyperactivity disorder are frequently described in contemporary literature. [23–27] Intricate relationship between the presence of tics, Tourette’s syndrome, and attention-deficit/hyperactivity disorder with a positive family history of obsessive-compulsive disorder (OCD), again speaks in favor of allelic heterogeneity/cross-disorder and puts forth the idea of the shared anatomical and pathobiological basis of some neurological and psychiatric disorders. [28,29]

A myriad of neurological manifestations characterize most neurometabolic diseases and inborn errors of metabolism. However, the burden of cognitive disabilities since early childhood is the most noticeable, striking, and constant feature. Childhood neurodevelopment disorders usually have a strong genetic predisposition. The parents of these children also have an increased chance of suffering from neurological diseases (similar or different neuropsychiatric disorders due to allelic heterogeneity/pleiotropy), harboring some neurological or psychiatric ailments that may have a significant impact on cognition and behavior. [30–33] These further fuel the neurological and psychopathological issues in the families. This coupled with disrupted thought processes might have complex relationships with faulty parenting and inappropriate health-care-seeking behaviors. [30–33]

### Social contributions to the genesis of difficult understanding and misconceptions:

Behavioral issues have been intricately intertwined in neurology, and their expression in families is varied and common. This is why a family with neurological diseases may also harbor truncated psychological issues. However, most of the time it remains uncared for, unattended, and undetected. This further increases the silent burden of psychological issues in these families and community at large, resulting in a change in disease perception, a faulty basic understanding of diseases, taboos, myths, and metacognitive biases which make the “road to health” full of obstacles. [34,35] This has been further coupled with inadequate/inappropriate healthcare infrastructures, “medical touts”, “quacks” (indigenous medical practitioners), corruption, and “infodemic”. All of them in combination make the “brain” fertile for the development of taboos, misconceptions, misbeliefs, metacognitive biases, and myths, which in turn, deters the development of appropriate health-seeking behavior. [36–38]

Most of the time, degenerative and irreversible diseases further complicate the issue. The genetically, structurally, or functionally compromised brain is predisposed to acquired brain diseases. [39,40] Several acquired neurological diseases have been observed in greater frequency among patients with genetically or structurally compromised brains and a history of hypoxic-ischemic encephalopathy (HIE). [39–41] HIE has been commonly considered as one of the dominant markers of inappropriate health awareness, poor health care delivery system, and poverty. [40–42] But in a real sense, poor health awareness/health-seeking behavior, faulty healthcare delivery, and poverty stem from a failure of the education system and a lack of higher cognitive exercising abilities in daily life. [43,44] Cognitive distortions and metacognitive biases in our society also have a crucial role to play in this regard. [45] Assumptions of the occurrence of acquired neurological ailments in patients with preexisting/background behavioral issues and disruptive cognitive functions are not far-fetched. This evidenced by the diseases with initial psychological/behavioral issues later mingled with neurological ailments, reflecting and at the same time enforcing the basic need for understanding of the complete spectrum of the disease under neuropsychiatric conundrum. [46–49] Families with genetically triggered psychological issues have every chance of heralding neurological ailments and vice versa. An overlapping spectrum of neurological and psychiatric issues harboring several genetic correlations underneath, only visible by its finer sense, reflects the two sides of a similar disease conundrum. [45–49]

### Pitfalls in perception

Contemporary studies revealed that neuroticism in early life could have an intriguing relationship with the development of Alzheimer’s and Parkinson’s diseases later. [50–52] Neuroticism, also believed to affect Alzheimer’s disease neuropathology, may be indicative of differential and the diverse manifestation of the same disease continuum sequestered temporally. [50–53] Behavioral manifestations of neurological ailments may be disseminated over time and, at the same time, may impact primary disease as well. [53] Overlap behavioral symptoms with so-called “organic” neurological ailments have long been underestimated. Behavioral issues among the close family members of patients with neurological disease influence the disease perception, understanding of the disease, and related healthcare-seeking attitude. [54,55] In our experience, the faulty perception of the disease manifestation in the developing/underdeveloped regions of the world lead to the need for multiple health-seeking attitude; this often leads to biased and misleading counterintuitive and counterproductive opinions, which pave the path for a huge economic burden on the community and reinforce unethical “medical business”. [54]

Low educational attainment has been thought to act as the foundation-stone for poverty. This, again, is thought to stem from disruptive cognitive abilities, which, unfortunately, herald generations with low cognitive abilities, cognitive biases, metacognitive distortions in mind, and economic burden on the shoulder. [54–57] All of these form a vicious cycle and predispose to an ever-increasing poverty and economic crisis, subsequently leading to disabled families and society, constituting the basis of human suffering. [54–58]

The natural history of diseases coupled with misguidance from the medical fraternity, augment of misbeliefs, misconceptions, and myths, and further fortify each other firmly borrowing an example, a demyelinating CNS disease can have a spontaneous recovery; however, if incidentally that spontaneous recovery coincides with a treatment with alternative medicine or some treatment based on “pseudoscience”, it will give rise to a faulty perception. This misperception gets stored permanently inside the brain of family members/caregivers who, by default, are also genetically predisposed to have some neuropsychiatric ailments, either full-blown or in its truncated forms, leading to the formation of a hub of misconceptions and metacognitive biases within the families, which usually get “viral” viciously through the porous avenues of social media. [59]

Overindulgence, common in families with neuropsychiatric diseases, is considered one of the components of behavioral addiction, which usually gives rise to myths, metacognitive biases, and superstitions. [59–67] Patients with neuropsychiatric ailments with low cognitive abilities, behavioral issues, physical limitations, and low conscientiousness levels are likely to be taken advantage of and misdirected by persons with low conscientiousness levels but with relatively better cognitive abilities. This contributes majorly to align the threads of addiction network (behavioral addiction and substance use disorders) and organized crime together.[59–68] Furthermore, hyperreligiosity and over-ritualistic behaviors (as a part of the symptom complex of OCD) extend their hands to bolster up “pseudo beliefs”, misconceptions, and superstitions. [69–76] Every minor detail and important scientific observation is always exigent to be registered and must be adequately substantiated through statistics. However, it may not be always possible to substantiate statistically, but that does not make these observations wrong or unworthy. [77–79] Contrary to the contemporary research approach, it is going to be onerous to perceive, interpret and determine the degree and severity of disruption of the default mode network (the “social brain”), misconceptions, and metacognitive distortions which ruin the society/community at large. [76] Due to lack of objective methods for measuring these parameters, these observations remain untold. However, one can also not deny its existence. Rather, here the authors intend to say with affirmation that statistical jugglery often misses the finer interpretations and observations, which is responsible for misinterpretation and faulty appreciation of the current scenario and may cause harm to the healthcare system in developing countries. [77–79] Aimless endeavor to quantify and analyze qualitative data further complicates the state of affairs. Issues of developing low- and middle-income countries are different from those of developed countries. [77–86] The maladaptive appraisal, high regard towards an “undeserving” indigenous practitioners/medical professional or medical institution(s) shown by the general people with low educational attainment and low cognitive reserve, “pseudo beliefs” or with OCD, often result in “pseudo inflation” of reputation/ego among the persons concerned. [87–95] These form the basis of several loopholes in identifying appropriate medical professionals/institutions to be consulted, resulting in the development of a “GOD-complex” among some professionals, casting a huge economic burden on the individuals or society at large and have the potential to mislead the entire ailing community. [87–95] Poor educational background secondary to low cognitive reserve, behavioral issues due to co-clustering of neurological ailments, and poor perception and basic understanding of disease lead to “stigmatization” of several diseases, negatively impacting the healthcare-seeking behavior of the community. [87–95]

“History taking”, the “forgotten skill” with the unique potential to diagnose several neurological ailments, has been submerged into oblivion. Medical professionals are thus compelled to be inclined towards the (in the light of inappropriate appraisal of history) current costlier sophisticated investigations without adequate yield, putting a huge economic burden on the frail community. [77–79] The entire scenario stems from improper/vague disclosure of history by the family members of patients with neurological ailments, in most cases, arising from the poor perception of disease symptomatologies and low educational attainment. Although, in the minority of cases, proper history elicitation on the part of medical professionals appears to be faulty, both increase the suffering of the ailing community exponentially. For example, caregivers blatantly denying any family history of neuropsychiatric illness occurs quite commonly in our daily practice. In our lifetime perception, this is often absolutely erroneous, as most neurological and psychiatric diseases are not sporadic in the true sense. However, the contradictory notion prevails probably due to either erroneous historical interpretation, poor perception about the ‘disease continuum’ (among both caregivers and sometimes consultants’ level), or failure to appreciate different faces of the same disease; for example, the effect of “multifactorial inheritance” and the role of multiple genes playing simultaneously for a specific disease like schizophrenia, depression, or Alzheimer’s disease. Family history of neuropsychiatric disorders is hardly believed to be negative as several association studies have already established the crucial relationship of parental psychopathology in patients with epilepsy, autistic spectrum disorder, attention deficit hyperkinetic disorder, and tics. [96–106]

### Parental psychopathology

The association of parental psychopathology in neurometabolic (e.g., Wilson’s disease) and neuroimmunological diseases (including demyelinating diseases of CNS) are yet to be established. [107–114] However, our lifetime observations have revealed various psychopathologies among parents/caregivers that are not merely explained by depression and anxiety related to the sufferings of their children/closer ones from severe neurological ailments. This might probably be arising from the truncated or partial effect(s) of the same genetic mutation(s) their children are harboring, resulting in the expression of underestimated behavioral counterpart of the same disease conundrum. [107–114] The same applies to various neurodegenerative diseases.

The authors have experienced that even in neurodegenerative dementias like Alzheimer’s disease, frontotemporal dementia, and dementia associated with Parkinson’s disease, there is a plethora of psychiatric manifestations (evident from retrospective history analysis) among patients having the diseases, i.e., neurodegenerative dementias, as well as among their first-degree relatives. This only firmly postulates that neurodegenerative diseases have their pathogenetic clue, potentially hidden much upstream, and can have either isolated neurological or psychiatric manifestations or expression of disease through the plane of neuropsychiatric convergence. [107–114] The strong association between neuroticism and the development of Alzheimer’s disease in later life also holds the notion of neuropsychiatric convergence firmly. It can be extrapolated to any disease entity started in either way and finally merged into a neuropsychiatric continuum. [50–52] This further explains that the same genetic abnormality or multifactorial inheritance can have a specific disease with diverse and varied phenotypes. Herein, the authors intend to draw the diagram of the complete neuropsychiatric loop rather than focus upon only neural or psychic components separately, which can result in a skewed interpretation and under-recognition of the entire aspect of any neurological or psychiatric disease entity.

Psychopathologies of first-degree family members of degenerative or genetic neurological or psychiatric disease(s), which authors hypothesize to stem from a truncated or erratic representation of similar genetic perturbation(s) coupled with environmental influences (i.e., multifactorial inheritance and epigenetics), potentially pave the path for poor cognitive abilities, lower educational attainments, higher-order perceptual difficulties, defective sensory processing, disorders in appreciation of environmental influences, the surge of myths, misconceptions, magical beliefs, mass-hysteria, metacognitive biases as well as distortions, and stigmatizations. These issues get further complicated by poverty and unemployment and evolve around consanguinity, the harbinger of cumulative neuropsychiatric disease burden within the family. [107–114]

### Loopholes in medical education

The shared pathophysiological basis of neurological and psychiatric ailments often manifests with truncated neuropsychiatric manifestations (i.e., only behavioral symptoms), leading to the cumulative burden of neuropsychiatric illness in the family. These are coupled and interrelated with low cognitive abilities, poverty, unemployment, consanguinity, magical beliefs, misconceptions, myths, mass hysteria, herd behaviors, metacognitive bias, and distortions, social deprivation, lack of proper guidance and infrastructure, stigmatizations, the devastating natural course of degenerative diseases in association with wrong perception and understanding of disease(s) evolve around the sufferings of the human being with neurological ailments. [115–120] Common neurological diseases like stroke, epilepsy, demyelinating disorders, immunologically mediated diseases, neurometabolic diseases, and degenerative diseases have strong genetic bases. [107–120] Family history of similar illness may be absent. However, if asked specifically, other neurological or psychiatric ailments in close family members often indicate an underlying crucial and intricate genetic basis with diverse disease phenotype(s). [107–121] Most noteworthy in this notion is that even neuroinfectious diseases have a strong family history of neurological and psychiatric ailments if carefully looked into, indicating that brain has to be genetically compromised even for neuroinfection.

The cumulative neuropsychiatric and consequent economic burden on family and society largely become manifold by the manipulative claws of “corruption” and “infodemic” known to thrive on spreading misconceptions and mistrust against the medical fraternity. [122–125] “Human sufferings” toughens its roots by breach of beliefs in the doctor-patient relationship, primed by mistrust, misconception, poor perception about diseases, and faulty stress-coping strategies among caregivers. Poor perception about diseases, lack of understanding, disruptive cognitive abilities, idiosyncratic beliefs, distortions in history deliberation, and inability to express exact problems stemming from low educational levels all eventually lead to excessive dependence on the irrational cascade of investigations and interventions, leaving the enormous economic burden on the already-frail community with neuropsychiatric diseases continuum, in association with disrupted cognitive abilities and reserve. [122–134]

Patterns of basic medical education (graduate) and higher medical education (post-graduate and post-doctorate) in developing countries have flaws that directly and indirectly enhance suffering. In most institutions of developing countries, there is little room for combined neurology and psychiatry training for residents resulting in a truncated/skewed knowledge base about diseases of the same conundrum/spectrum leading to frequent misdiagnosis, over-dependence on investigations, and polypharmacy as well as therapeutic misadventures. [134–146]

Questions may arise about whether an accurate diagnosis of advanced neurodegenerative or irreversible diseases is essential, as the prognosis remains the same even after an accurate diagnosis. However, accurate diagnosis is essential and of utmost importance 1) to curtail caregivers’ stress; 2) to limit the need for multiple consultancies; 3) to establish appropriate coping strategies; 4) to get rid of huge economic burden due to multiple consultancies; 5) to decrease the need for admission and irrelevant investigations, and last but not least; and 6) to raise voice against myths, misconception, mistrust, misbeliefs, and social stigma. Furthermore, the irreversible nature of diseases frequently has an underpinning genetic basis, which, if identified in the same due course of the diseases, also helps in genetic and premarital counseling to decrease the transgenerational spread of irreversible genetic diseases, which will eventually decrease neuropsychiatric disease burden and cognitive disabilities in families as well as in society. [147–154]

In most developing countries, psychiatric ailments and various neurological illnesses like epilepsy have enormous social stigma, and people with these diseases are ostracized at a large scale by society due to poor perception, awareness, and low education. [155–161] Family members of patients with psychiatric illnesses, as well as patients with epilepsy, often try to hide their history due to fear of being social outcast and many times due to their cognitive disabilities (probable causes of which have been discussed earlier) and poor perception and knowledge about the diseases, results in further complications and misdiagnosis. [157–163] Faulty healthcare-seeking behavior and attitude often leads to the inappropriate selection of “specialists” and makes room for quacks, alternative medicine, and “pseudoscience” to flourish, which further strengthens the taboos, stigma, and suffering. [155–163]

Authors’ experience in neurology out-patient departments (OPDs) unveiled that patients having pure somatoform pain disorders, somatization, depression, obsessive-compulsive spectrum disorders, and dissociative conversion disorder share the major percentage of daily OPD visitors (poor execution of referral system) and are also being satisfied by knowing that they have “nerve problems”. These patients become disappointed on disclosing that they have psychiatric issues and need to get treatment from psychiatrists and are not ready to accept even if counseled correctly. [155–161] On the other hand, authors have also found a good number of patients with epilepsy, “organic” psychosis, and “organic” mood disorders in psychiatry OPDs of other tertiary-care hospitals (ongoing study by same authors; data unpublished).

As mentioned, due to lack of integrated training in neuropsychiatry frequently patients with psychiatric ailments are being partially, incompletely and inefficiently treated by neurologists. The behavioral presentations of “organic” systemic and neurological diseases like systemic lupus erythematosus psychosis, autoimmune encephalitis, Wernicke’s aphasia, Wernicke’s encephalopathy, Wilson disease, behavior variant of frontotemporal dementia, Alzheimer’s dementia, Huntington’s disease, non-motor presentation of Parkinson’s disease, diffuse Lewy-body disease, episodic presentations of Moyamoya angiopathy, transient ischemic attack, multiple sclerosis, complex partial seizures (non-motor, behavior and cognitive arrest variants), sub-acute sclerosing panencephalitis, and progressive myoclonic epilepsies are frequently being misdiagnosed by psychiatrists, results in series of investigations, multiple consultations, frequent hospital admission, need for polypharmacy, economic burden and enormous sufferings of patients and family members. [135–154]

### Future directions

Endeavor to cut short the sufferings, poverty and economic burden of families with neuropsychiatric ailments must include: 1) raising scientific voice against consanguinity to break the chain of exponential expression of pathogenic gene in the families; 2) wherever appropriate, even in resource-poor developing countries, provision for genetic testing and genetic counseling should be offered; 3) robust infrastructure of free education for all children should be the primary notion and must be executed with vigilant higher-authority supervision (because education is the backbone behind the development of any nation, and children are the building blocks of any nation in making); 4) strict vigilance and legislations to be ensured against misdirected, myths and misconception-based health news in electronic, printing and social medias; 5) promotion of positive education-based mass-campaign programs, with the help of the “mass leaders” against taboos, misbeliefs and stigma about neuropsychiatric ailments to be organized at regular intervals under supervision of concerned committees; 6) people must be encouraged to share their mental issues freely with family members and with psychiatrists through health education, and at the same time, they should be warned about possible disastrous outcome of suppression; 7) indigenous medical practitioners are needed to be educated and trained enough before declaring them capable of dealing with neuropsychiatric ailments (if at all this is necessary in rural poverty-stricken pockets of low- and middle-income countries); 8) unethical polypharmacy, irrational investigations as well as any vested interest other than patients’ benefit must be condemned and prohibited authoritatively; and lastly 9) integrated and comprehensive training program for neurology and psychiatry residents must be instituted at the earliest in all developing low- and middle-income countries to overcome the skew-deviation in patients’ diagnosis, and treatment, with an aim that it would potentially mitigate sufferings, alleviate economic burden and cognitive disabilities of families with neuropsychiatric diseases.

## Figures and Tables

**Figure:**
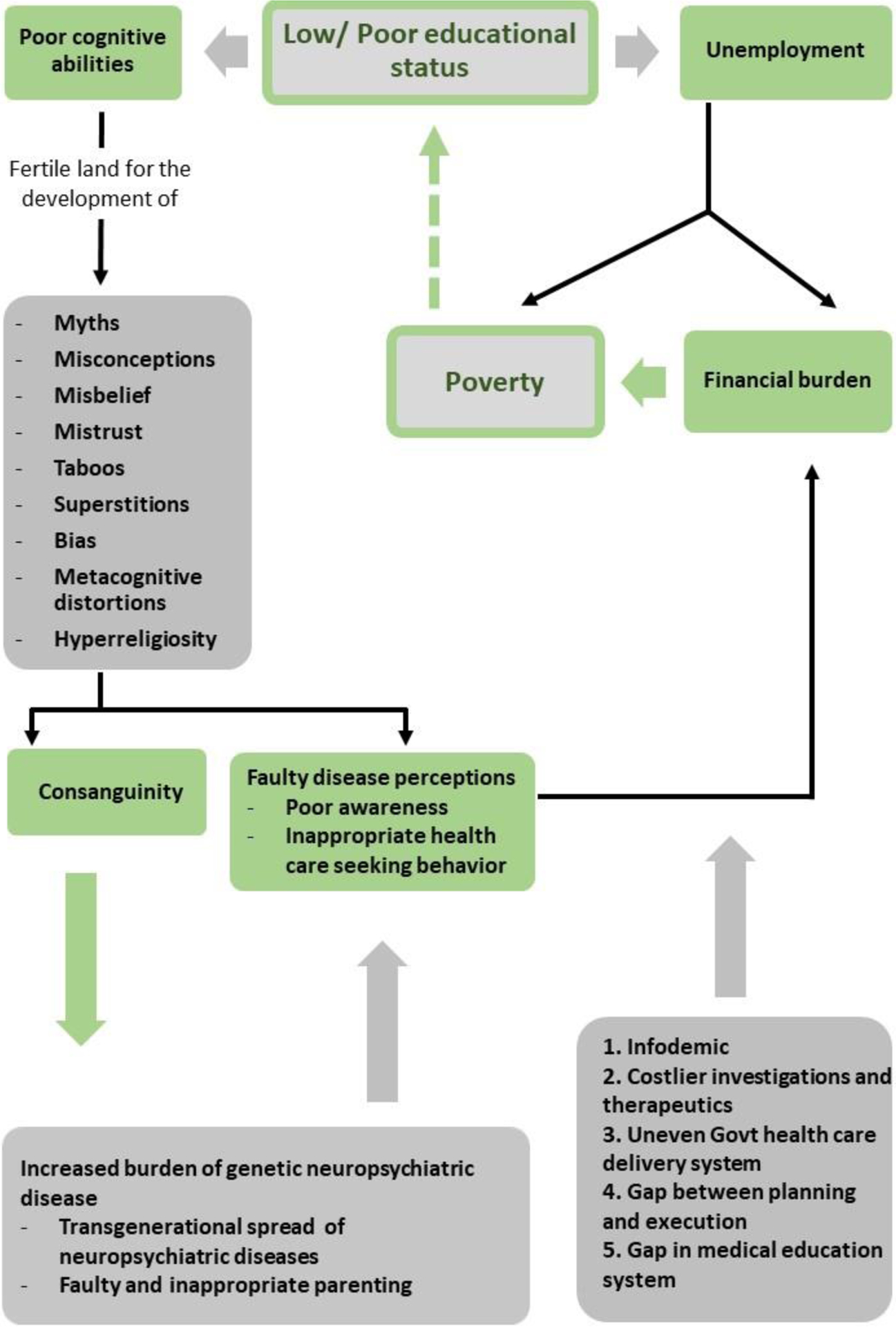
Flowchart underpinning the neuropsychological basis of poverty and human suffering.

## Data Availability

No datasets were analyzed or generated during the current study as it is a theory-based article.

## References

[1] Hoehe MR, Morris-Rosendahl DJ. The role of genetics and genomics in clinical psychiatry. Dialogues Clin Neurosci. 2018;20(3):169–177. doi:10.31887/DCNS.2018.20.3/mhoehe 30581286 PMC6296395

[2] Toft M Advances in genetic diagnosis of neurological disorders. Acta Neurol Scand Suppl. 2014;(198):20–25. doi:10.1111/ane.12232 24588502

[3] Dichgans M, Pulit SL, Rosand J. Stroke genetics: discovery, biology, and clinical applications. Lancet Neurol. 2019;18(6):587–599. doi:10.1016/S1474-4422(19)30043-2 30975520

[4] Weber YG, Biskup S, Helbig KL, Von Spiczak S, Lerche H. The role of genetic testing in epilepsy diagnosis and management. Expert Rev Mol Diagn. 2017;17(8):739–750. doi:10.1080/14737159.2017.1335598 28548558

[5] Axisa PP, Hafler DA. Multiple sclerosis: genetics, biomarkers, treatments. Curr Opin Neurol. 2016;29(3):345–353. doi:10.1097/WCO.0000000000000319 27058221 PMC7882195

[6] Coffey AJ, Durkie M, Hague S, A genetic study of Wilson’s disease in the United Kingdom. Brain. 2013;136(Pt 5):1476–1487. doi:10.1093/brain/awt035 23518715 PMC3634195

[7] Wamsley B, Geschwind DH. Functional genomics links genetic origins to pathophysiology in neurodegenerative and neuropsychiatric disease. Curr Opin Genet Dev. 2020;65:117–125. doi:10.1016/j.gde.2020.05.032 32634676 PMC8171040

[8] Finkbeiner S Functional genomics, genetic risk profiling and cell phenotypes in neurodegenerative disease. Neurobiol Dis. 2020;146:105088. doi:10.1016/j.nbd.2020.105088 32977020 PMC7686089

[9] Sudhakar V, Richardson RM. Gene Therapy for Neurodegenerative Diseases. Neurotherapeutics. 2019;16(1):166–175. doi:10.1007/s13311-018-00694-0 30542906 PMC6361055

[10] Bertram L, Tanzi RE. The genetic epidemiology of neurodegenerative disease. J Clin Invest. 2005;115(6):1449–1457. doi:10.1172/JCI24761 15931380 PMC1137006

[11] Kovacs GG. Concepts and classification of neurodegenerative diseases. Handb Clin Neurol. 2017;145:301–307. doi:10.1016/B978-0-12-802395-2.00021-3 28987178

[12] Anaz S, Maddirevul S, Salpietr V, . Expanding the genetic heterogeneity of intellectual disability [published correction appears in Hum Genet. 2017 Dec 29;:]. Hum Genet. 2017;136(11–12):1419–1429. doi:10.1007/s00439-017-1843-2 28940097

[13] Han B, Pouget JG, Slowikowski K, A method to decipher pleiotropy by detecting underlying heterogeneity driven by hidden subgroups applied to autoimmune and neuropsychiatric diseases. Nat Genet. 2016;48(7):803–810. doi:10.1038/ng.3572 27182969 PMC4925284

[14] Pichet Binette A, Vachon-Presseau É, Morris J, Amyloid and Tau Pathology Associations With Personality Traits, Neuropsychiatric Symptoms, and Cognitive Lifestyle in the Preclinical Phases of Sporadic and Autosomal Dominant Alzheimer’s Disease. Biol Psychiatry. 2021;89(8):776–785. doi:10.1016/j.biopsych.2020.01.023 32228870 PMC7415608

[15] Greenberg DR, Khandwala YS, Lu Y, Stevenson DK, Shaw GM, Eisenberg ML. Disease burden in offspring is associated with changing paternal demographics in the United States. Andrology. 2020;8(2):342–347. doi:10.1111/andr.12700 31478609

[16] Everett Y, Martin CG, Zalewski M. A Systematic Review Focusing on Psychotherapeutic Interventions that Impact Parental Psychopathology, Child Psychopathology and Parenting Behavior. Clin Child Fam Psychol Rev. 2021;24(3):579–598. doi:10.1007/s10567-021-00355-3 34254219 PMC8970553

[17] Calkins SD, Propper C, Mills-Koonce WR. A biopsychosocial perspective on parenting and developmental psychopathology. Dev Psychopathol. 2013;25(4 Pt 2):1399–1414. doi:10.1017/S0954579413000680 24342847

[18] Hartman L, Jenkinson C, Morley D. Young People’s Response to Parental Neurological Disorder: A Structured Review. Adolesc Health Med Ther. 2020;11:39–51. Published 2020 Mar 26. doi:10.2147/AHMT.S237807 32273785 PMC7105371

[19] Rodenburg R, Meijer AM, Scherphof C, Parenting and restrictions in childhood epilepsy. Epilepsy Behav. 2013;27(3):497–503. doi:10.1016/j.yebeh.2013.01.026 23602224

[20] Huber-Mollema Y, Oort FJ, Lindhout D, Rodenburg R. Well-being of mothers with epilepsy with school-aged children. Epilepsy Behav. 2020;105:106966. doi:10.1016/j.yebeh.2020.106966 32146338

[21] Rodenburg R, Meijer AM, Deković M, Aldenkamp AP. Family factors and psychopathology in children with epilepsy: a literature review. Epilepsy Behav. 2005;6(4):488–503. doi:10.1016/j.yebeh.2005.03.006 15907744

[22] Adewuya AO. Parental psychopathology and self-rated quality of life in adolescents with epilepsy in Nigeria. Dev Med Child Neurol. 2006;48(7):600–603. doi:10.1017/S0012162206001253 16780631

[23] Xie S, Karlsson H, Dalman C, Family History of Mental and Neurological Disorders and Risk of Autism. JAMA Netw Open. 2019;2(3):e190154. Published 2019 Mar 1. doi:10.1001/jamanetworkopen.2019.0154 30821823 PMC6484646

[24] Romero-Gonzalez M, Chandler S, Simonoff E. The relationship of parental expressed emotion to co-occurring psychopathology in individuals with autism spectrum disorder: A systematic review. Res Dev Disabil. 2018;72:152–165. doi:10.1016/j.ridd.2017.10.022 29156388

[25] Thomas PA, King JS, Mendelson JL, Nelson-Gray RO. Parental psychopathology and expectations for the futures of children with autism spectrum disorder. J Appl Res Intellect Disabil. 2018;31(1):98–105. doi:10.1111/jar.12337 28205369

[26] Midouhas E, Yogaratnam A, Flouri E, Charman T. Psychopathology trajectories of children with autism spectrum disorder: the role of family poverty and parenting. J Am Acad Child Adolesc Psychiatry. 2013;52(10):1057–1065.e1. doi:10.1016/j.jaac.2013.07.011 24074472

[27] Propper L, Sandstrom A, Rempel S, Attention-deficit/hyperactivity disorder and other neurodevelopmental disorders in offspring of parents with depression and bipolar disorder [published online ahead of print, 2021 Jun 18]. Psychol Med. 2021;1–8. doi:10.1017/S0033291721001951 34140050

[28] Yu D, Mathews CA, Scharf JM, Cross-disorder genome-wide analyses suggest a complex genetic relationship between Tourette’s syndrome and OCD. Am J Psychiatry. 2015;172(1):82–93. doi:10.1176/appi.ajp.2014.13101306 25158072 PMC4282594

[29] Brem S, Grünblatt E, Drechsler R, Riederer P, Walitza S. The neurobiological link between OCD and ADHD. Atten Defic Hyperact Disord. 2014;6(3):175–202. doi:10.1007/s12402-014-0146-x 25017045 PMC4148591

[30] Thapar A, Cooper M, Rutter M. Neurodevelopmental disorders. Lancet Psychiatry. 2017;4(4):339–346. doi:10.1016/S2215-0366(16)30376-5 27979720

[31] Sudre G, Frederick J, Sharp W, Mapping associations between polygenic risks for childhood neuropsychiatric disorders, symptoms of attention deficit hyperactivity disorder, cognition, and the brain. Mol Psychiatry. 2020;25(10):2482–2492. doi:10.1038/s41380-019-0350-3 30700802 PMC6667324

[32] Adedokun ST, Yaya S Factors influencing mothers’ health care seeking behaviour for their children: evidence from 31 countries in sub-Saharan Africa. BMC Health Serv Res. 2020;20(1):842. Published 2020 Sep 7. doi:10.1186/s12913-020-05683-8 32894107 PMC7487813

[33] Currie G, Szabo J. Social isolation and exclusion: the parents’ experience of caring for children with rare neurodevelopmental disorders. Int J Qual Stud Health Well-being. 2020;15(1):1725362. doi:10.1080/17482631.2020.1725362 32048917 PMC7034477

[34] Taylor JJ, Williams NR, George MS. Beyond neural cubism: promoting a multidimensional view of brain disorders by enhancing the integration of neurology and psychiatry in education. Acad Med. 2015;90(5):581–586. doi:10.1097/ACM.0000000000000530 25340364 PMC4405399

[35] Benjamin S Neuropsychiatry and neural cubism. Acad Med. 2015;90(5):556–558. doi:10.1097/ACM.0000000000000531 25340368

[36] Bandstra NF, Camfield CS, Camfield PR. Stigma of epilepsy. Can J Neurol Sci. 2008;35(4):436–440. doi:10.1017/s0317167100009082 18973059

[37] Bou Nasif M, Koubeissi M, Azar NJ. Epilepsy - from mysticism to science. Rev Neurol (Paris). 2021;177(9):1047–1058. doi:10.1016/j.neurol.2021.01.021 34218946

[38] Javed A, Lee C, Zakaria H, Reducing the stigma of mental health disorders with a focus on low- and middle-income countries. Asian J Psychiatr. 2021;58:102601. doi:10.1016/j.ajp.2021.102601 33611083

[39] Whelan CD, Altmann A, Botía JA, Structural brain abnormalities in the common epilepsies assessed in a worldwide ENIGMA study. Brain. 2018;141(2):391–408. doi:10.1093/brain/awx341 29365066 PMC5837616

[40] Mora P, Hollier PL, Guimbal S, Blood-brain barrier genetic disruption leads to protective barrier formation at the Glia Limitans. PLoS Biol. 2020;18(11):e3000946. Published 2020 Nov 30. doi:10.1371/journal.pbio.3000946 33253145 PMC7728400

[41] Torres-Muñoz J, Fonseca-Perez JE, Laurent K. Biological and Psychosocial Factors, Risk Behaviors, and Perinatal Asphyxia in a University Hospital: Matched Case-Control Study, Cali, Colombia (2012–2014). Front Public Health. 2021;9:535737. Published 2021 Jun 21. doi:10.3389/fpubh.2021.535737 34235127 PMC8255785

[42] Jahan S Poverty and infant mortality in the Eastern Mediterranean region: a meta-analysis. J Epidemiol Community Health. 2008;62(8):745–751. doi:10.1136/jech.2007.068031 18621962

[43] Schiariti V, Simeonsson RJ, Hall K. Promoting Developmental Potential in Early Childhood: A Global Framework for Health and Education. Int J Environ Res Public Health. 2021;18(4):2007. Published 2021 Feb 19. doi:10.3390/ijerph18042007 33669588 PMC7923196

[44] Ho-Wai So S, Hoi-Kei Chan G, Kit-Wa Wong C, A randomised controlled trial of metacognitive training for psychosis, depression, and belief flexibility. J Affect Disord. 2021;279:388–397. doi:10.1016/j.jad.2020.09.126 33099054

[45] Hoge SK, Appelbaum PS. Ethics and neuropsychiatric genetics: a review of major issues. Int J Neuropsychopharmacol. 2012;15(10):1547–1557. doi:10.1017/S1461145711001982 22272758 PMC3359421

[46] Loi S, Chiu E. Witchcraft and Huntington’s disease: a salutary history of societal and medical stigmatisation. Australas Psychiatry. 2012;20(5):438–441. doi:10.1177/1039856212459587 23014122

[47] Smoller JW, Andreassen OA, Edenberg HJ, Faraone SV, Glatt SJ, Kendler KS. Psychiatric genetics and the structure of psychopathology [published correction appears in Mol Psychiatry. 2018 Mar 14;:]. Mol Psychiatry. 2019;24(3):409–420. doi:10.1038/s41380-017-0010-4 29317742 PMC6684352

[48] Ahmed HM, Advani R, Arif AA, Khan S. An Assessment of the Knowledge, Attitudes, and Practices of Patients and Families with Diagnoses of Hereditary Neuromuscular Disorders. Neuroepidemiology. 2020;54(3):265–271. doi:10.1159/000505330 32018248

[49] Mayor R, Gunn S, Reuber M, Simpson J. Experiences of stigma in people with epilepsy: A meta-synthesis of qualitative evidence. Seizure. 2022;94:142–160. doi:10.1016/j.seizure.2021.11.021 34915348

[50] Terracciano A, Aschwanden D, Stephan Y, Neuroticism and Risk of Parkinson’s Disease: A Meta-Analysis. Mov Disord. 2021;36(8):1863–1870. doi:10.1002/mds.28575 33817817 PMC8376751

[51] Terracciano A, Sutin AR. Personality and Alzheimer’s disease: An integrative review. Personal Disord. 2019;10(1):4–12. doi:10.1037/per0000268 30604979 PMC6345278

[52] Terracciano A, Aschwanden D, Passamonti L, Is neuroticism differentially associated with risk of Alzheimer’s disease, vascular dementia, and frontotemporal dementia?. J Psychiatr Res. 2021;138:34–40. doi:10.1016/j.jpsychires.2021.03.039 33819874 PMC8192471

[53] Dubey S, Dubey MJ, Ghosh R, Mukherjee D, Pandit A, Benito-León J. Behavioral and psychological symptoms in neurodegenerative dementias: harbinger, follower, or constant collateral?. Egypt J Neurol Psychiatr Neurosurg. 2022;58:102. doi:10.1186/s41983-022-00538-x 36160603 PMC9503106

[54] Lazar M, Davenport L. Barriers to Health Care Access for Low Income Families: A Review of Literature. J Community Health Nurs. 2018;35(1):28–37. doi:10.1080/07370016.2018.1404832 29323941

[55] Patel V, Kleinman A. Poverty and common mental disorders in developing countries. Bull World Health Organ. 2003;81(8):609–615. 14576893 PMC2572527

[56] Nieuwenhuis J, Kleinepier T, van Ham M. The Role of Exposure to Neighborhood and School Poverty in Understanding Educational Attainment. J Youth Adolesc. 2021;50(5):872–892. doi:10.1007/s10964-021-01427-x 33829400 PMC8043918

[57] Porterfield SL, McBride TD. The effect of poverty and caregiver education on perceived need and access to health services among children with special health care needs. Am J Public Health. 2007;97(2):323–329. doi:10.2105/AJPH.2004.055921 17194872 PMC1781389

[58] Barnish MS, Tan SY, Taeihagh A, Tørnes M, Nelson-Horne RVH, Melendez-Torres GJ. Linking political exposures to child and maternal health outcomes: a realist review. BMC Public Health. 2021;21(1):127. Published 2021 Jan 12. doi:10.1186/s12889-021-10176-2 33435933 PMC7802227

[59] Kuss DJ, Griffiths MD. Online social networking and addiction--a review of the psychological literature. Int J Environ Res Public Health. 2011;8(9):3528–3552. doi:10.3390/ijerph8093528 22016701 PMC3194102

[60] Kwak MJ, Cho H, Kim DJ. The Role of Motivation Systems, Anxiety, and Low Self-Control in Smartphone Addiction among Smartphone-Based Social Networking Service (SNS) Users. Int J Environ Res Public Health. 2022;19(11):6918. Published 2022 Jun 5. doi:10.3390/ijerph19116918 35682501 PMC9180772

[61] Hudson G, Jansli SM, Erturk S, Investigation of Carers’ Perspectives of Dementia Misconceptions on Twitter: Focus Group Study. JMIR Aging. 2022;5(1):e30388. Published 2022 Jan 24. doi:10.2196/30388 35072637 PMC8822432

[62] Patil AU, Madathil D, Huang CM. Age-related and individual variations in altered prefrontal and cerebellar connectivity associated with the tendency of developing internet addiction. Hum Brain Mapp. 2021;42(14):4525–4537. doi:10.1002/hbm.25562 34170056 PMC8410527

[63] Sjölund S, Allebeck P, Hemmingsson T. Intelligence quotient (IQ) in adolescence and later risk of alcohol-related hospital admissions and deaths--37-year follow-up of Swedish conscripts. Addiction. 2012;107(1):89–97. doi:10.1111/j.1360-0443.2011.03544.x 21692890

[64] Bailey AJ, Gerst K, Finn PR. Intelligence moderates the relationship between delay discounting rate and problematic alcohol use. Psychol Addict Behav. 2020;34(1):175–181. doi:10.1037/adb0000471 31219266 PMC6923633

[65] Bart CP, Nusslock R, Ng TH, Decreased reward-related brain function prospectively predicts increased substance use. J Abnorm Psychol. 2021;130(8):886–898. doi:10.1037/abn0000711 34843292 PMC8634780

[66] Müller SM, Schiebener J, Brand M, Liebherr M. Decision-making, cognitive functions, impulsivity, and media multitasking expectancies in high versus low media multitaskers. Cogn Process. 2021;22(4):593–607. doi:10.1007/s10339-021-01029-2 34047893 PMC8547206

[67] Welte JW, Wieczorek WF. Alcohol, intelligence and violent crime in young males. J Subst Abuse. 1998;10(3):309–319. doi:10.1016/s0899-3289(99)00002-4 10689662

[68] Dubey MJ, Ghosh R, Chatterjee S, Biswas P, Chatterjee S, Dubey S. COVID-19 and addiction. Diabetes Metab Syndr. 2020;14(5):817–823. doi:10.1016/j.dsx.2020.06.008 32540735 PMC7282772

[69] Moulding R, Kyrios M. Anxiety disorders and control related beliefs: the exemplar of Obsessive-Compulsive Disorder (OCD). Clin Psychol Rev. 2006;26(5):573–583. doi:10.1016/j.cpr.2006.01.009 16647173

[70] Hezel DM, McNally RJ. A Theoretical review of cognitive biases and deficits in obsessive-compulsive disorder. Biol Psychol. 2016;121(Pt B):221–232. doi:10.1016/j.biopsycho.2015.10.012 26594019

[71] Olatunji BO, Christian C, Brosof L, Tolin DF, Levinson CA. What is at the core of OCD? A network analysis of selected obsessive-compulsive symptoms and beliefs. J Affect Disord. 2019;257:45–54. doi:10.1016/j.jad.2019.06.064 31299404

[72] Tümkaya S, Karadağ F, Yenigün EH, Özdel O, Kashyap H. Metacognitive Beliefs and Their Relation with Symptoms in Obsessive-Compulsive Disorder. Noro Psikiyatr Ars. 2018;55(4):358–363. doi:10.29399/npa.22655 30622394 PMC6300830

[73] Mathieu SL, Conlon EG, Waters AM, McKenzie ML, Farrell LJ. Inflated Responsibility Beliefs in Paediatric OCD: Exploring the Role of Parental Rearing and Child Age. Child Psychiatry Hum Dev. 2020;51(4):552–562. doi:10.1007/s10578-019-00938-w 31664631

[74] Pugh K, Luzon O, Ellett L. Responsibility beliefs and persecutory delusions. Psychiatry Res. 2018;259:340–344. doi:10.1016/j.psychres.2017.10.044 29120840

[75] Dar R, Kahn DT, Carmeli R. The relationship between sensory processing, childhood rituals and obsessive-compulsive symptoms. J Behav Ther Exp Psychiatry. 2012;43(1):679–684. doi:10.1016/j.jbtep.2011.09.008 21963890

[76] Dubey S, Dubey MJ, Ghosh R, The cognitive basis of psychosocial impact in COVID-19 pandemic. Does it encircle the default mode network of the brain? A pragmatic proposal. Med Res Arch. 2022;10(3):10.18103/mra.v10i3.2707. doi:10.18103/mra.v10i3.2707 PMC9071110 35530572

[77] Burgers JS. Opschudding over evidencebased medicine: van reductionisme naar realisme in de toepassing van richtlijnen [Criticism of evidence-based medicine: from reductionism to realism in the application of guidelines]. Ned Tijdschr Geneeskd. 2015;159:A8376. 25650035

[78] Kouimtsidis Ch, John-Smith S, Kemp P, Ikkos G Evidence based mental healthcare and service innovation: review of concepts and challenges. Psychiatriki. 2013;24(1):45–54. 23603268

[79] Rogers WA. Evidence based medicine and justice: a framework for looking at the impact of EBM upon vulnerable or disadvantaged groups. J Med Ethics. 2004;30(2):141–145. doi:10.1136/jme.2003.007062 15082806 PMC1733835

[80] Alemayehu C, Mitchell G, Nikles J. Barriers for conducting clinical trials in developing countries-a systematic review. Int J Equity Health. 2018;17(1):37. Published 2018 Mar 22. doi:10.1186/s12939-018-0748-6 29566721 PMC5863824

[81] Rahman MM, Ghoshal UC, Ragunath K, Biomedical research in developing countries: Opportunities, methods, and challenges. Indian J Gastroenterol. 2020;39(3):292–302. doi:10.1007/s12664-020-01056-5 32607962 PMC7325473

[82] Sumathipala A, Siribaddana S, Patel V. Under-representation of developing countries in the research literature: ethical issues arising from a survey of five leading medical journals. BMC Med Ethics. 2004;5:E5. Published 2004 Oct 4. doi:10.1186/1472-6939-5-5 15461820 PMC524359

[83] Emanuel EJ, Wendler D, Killen J, Grady C. What makes clinical research in developing countries ethical? The benchmarks of ethical research. J Infect Dis. 2004;189(5):930–937. doi:10.1086/381709 14976611

[84] Noor RA. Health research oversight in Africa. Acta Trop. 2009;112 Suppl 1:S63–S70. doi:10.1016/j.actatropica.2009.08.019 19698692

[85] Kanoute A, Faye D, Bourgeois D. Current status of oral health research in Africa: an overview. Int Dent J. 2012;62(6):301–307. doi:10.1111/j.1875-595X.2012.00123x 23252587 PMC9374928

[86] Sharan P, Gallo C, Gureje O, Mental health research priorities in low- and middle-income countries of Africa, Asia, Latin America and the Caribbean. Br J Psychiatry. 2009;195(4):354–363. doi:10.1192/bjp.bp.108.050187 19794206 PMC3432479

[87] Ghosh R, Roy D, Benito-León J Indigenous medical practitioners to curb coronavirus disease-19 crisis in India: a pragmatic proposal? Indian J Commun Med. 2022 doi: 10.4103/ijcm.ijcm_997_21. PMC11927834 40124804

[88] Upenieks L, Ford-Robertson J, Robertson JE. Trust in God and/or Science? Sociodemographic Differences in the Effects of Beliefs in an Engaged God and Mistrust of the COVID-19 Vaccine. J Relig Health. 2022;61(1):657–686. doi:10.1007/s10943-021-01466-5 34843011 PMC8628135

[89] Sharath S Doctors in India suffer from a God complex. Indian J Med Ethics. 2008;5(3):149. doi:10.20529/IJME.2008.054 18754242

[90] Cotton S, Puchalski CM, Sherman SN, Spirituality and religion in patients with HIV/AIDS [published correction appears in J Gen Intern Med. 2009 Aug;24(8):994]. J Gen Intern Med. 2006;21 Suppl 5(Suppl 5):S5–S13. doi:10.1111/j.1525-1497.2006.00642.x PMC1924778 17083501

[91] Klitzman R, Doctor, Will You Pray for Me? Responding to Patients’ Religious and Spiritual Concerns. Acad Med. 2021;96(3):349–354. doi:10.1097/ACM.0000000000003765 33003037 PMC7933033

[92] Superdock AK, Barfield RC, Brandon DH, Docherty SL. Exploring the vagueness of Religion & Spirituality in complex pediatric decision-making: a qualitative study. BMC Palliat Care. 2018;17(1):107. Published 2018 Sep 12. doi:10.1186/s12904-018-0360-y 30208902 PMC6134505

[93] Gross M Between Party, People, and Profession: The Many Faces of the ‘Doctor’ during the Cultural Revolution. Med Hist. 2018;62(3):333–359. doi:10.1017/mdh.2018.23 29886861 PMC6113761

[94] Dornan T, Roy Bentley S, Kelly M. Medical teachers’ discursive positioning of doctors in relation to patients. Med Educ. 2020;54(7):628–636. doi:10.1111/medu.14074 31991480 PMC7317436

[95] Szaflarski M, Ritchey PN, Leonard AC, Modeling the effects of spirituality/religion on patients’ perceptions of living with HIV/AIDS. J Gen Intern Med. 2006;21 Suppl 5(Suppl 5):S28–S38. doi:10.1111/j.1525-1497.2006.00646.x PMC1924787 17083497

[96] Freeman-Ferguso M. Tourette’s syndrome: challenging misconceptions and improving understanding. Nurs Child Young People. 2022;34(5):34–42. doi:10.7748/ncyp.2022.e1416 35312241

[97] Li M, Santpere G, Imamura Kawasawa Y, Integrative functional genomic analysis of human brain development and neuropsychiatric risks. Science. 2018;362(6420):eaat7615. doi:10.1126/science.aat7615 30545854 PMC6413317

[98] Ueda K, Black KJ. A Comprehensive Review of Tic Disorders in Children. J Clin Med. 2021;10(11):2479. Published 2021 Jun 3. doi:10.3390/jcm10112479 34204991 PMC8199885

[99] Kaddumukasa M, Smith PJ, Kaddumukasa MN, Epilepsy beliefs and misconceptions among patient and community samples in Uganda. Epilepsy Behav. 2021;114(Pt B):107300. doi:10.1016/j.yebeh.2020.107300 32758405

[100] Herrmann LK, Welter E, Berg AT, Perzynski AT, Van Doren JR, Sajatovic M. Epilepsy misconceptions and stigma reduction: Current status in Western countries. Epilepsy Behav. 2016;60:165–173. doi:10.1016/j.yebeh.2016.04.003 27208826 PMC6047062

[101] Grabowski DC, Fishman J, Wild I, Lavin B. Changing the neurology policy landscape in the United States: Misconceptions and facts about epilepsy. Health Policy. 2018;122(7):797–802. doi:10.1016/j.healthpol.2018.05.012 29908672

[102] Mohanty M, Beaulieu F, Sampath S, Tambunan D, Kataria S, Rosman NP. “Your Child Has Cerebral Palsy”: Parental Understanding and Misconceptions. J Child Neurol. 2021;36(8):648–654. doi:10.1177/0883073821991300 33620264

[103] Sansa E, Fray S, Jamoussi H, Knowledge and attitudes toward epilepsy among teachers in Tunisia. Epilepsy Behav. 2021;123:108260. doi:10.1016/j.yebeh.2021.108260 34481282

[104] Andersson K, Strang S, Zelano J, Chaplin J, Malmgren K, Ozanne A. Multiple stigma among first-generation immigrants with epilepsy in Sweden. Epilepsy Behav. 2021;115:107638. doi:10.1016/j.yebeh.2020.107638 33334721

[105] Cox JH, Nahar A, Termine C, Social stigma and self-perception in adolescents with tourette syndrome. Adolesc Health Med Ther. 2019;10:75–82. Published 2019 Jun 11. doi:10.2147/AHMT.S175765 31354374 PMC6573773

[106] Agha SS, Zammit S, Thapar A, Langley K. Maternal psychopathology and offspring clinical outcome: a four-year follow-up of boys with ADHD. Eur Child Adolesc Psychiatry. 2017;26(2):253–262. doi:10.1007/s00787-016-0873-y 27376657 PMC5306178

[107] Sellers R, Harold GT, Thapar A, Examining the Role of Genetic Risk and Longitudinal Transmission Processes Underlying Maternal Parenting and Psychopathology and Children’s ADHD Symptoms and Aggression: Utilizing the Advantages of a Prospective Adoption Design [published correction appears in Behav Genet. 2021 Jul;51(4):440]. Behav Genet. 2020;50(4):247–262. doi:10.1007/s10519-020-10006-y 32623545 PMC8286095

[108] Rusengamihigo D, Mutabaruka J, Biracyaza E, Magalakaki O, El’Husseini M. Parental mental illness and their offspring’s mental health in Rwanda: neuropsychiatric hospital of Rwanda. BMC Psychol. 2021;9(1):135. Published 2021 Sep 4. doi:10.1186/s40359-021-00633-3 34481518 PMC8418748

[109] Yu J, Patel RA, Gilman SE. Childhood disadvantage, neurocognitive development and neuropsychiatric disorders: Evidence of mechanisms. Curr Opin Psychiatry. 2021;34(3):306–323. doi:10.1097/YCO.0000000000000701 33587493 PMC9458466

[110] Al Jumah M, Al Malik Y, AlKhawajah NM, Family Planning for People with Multiple Sclerosis in Saudi Arabia: an Expert Consensus. Mult Scler Int. 2021;2021:6667006. Published 2021 Feb 15. doi:10.1155/2021/6667006 33628508 PMC7899766

[111] Johnson MR, Shorvon SD. Heredity in epilepsy: neurodevelopment, comorbidity, and the neurological trait. Epilepsy Behav. 2011;22(3):421–427. doi:10.1016/j.yebeh.2011.07.031 21890419

[112] Heinzen EL, Neale BM, Traynelis SF, Allen AS, Goldstein DB. The genetics of neuropsychiatric diseases: looking in and beyond the exome. Annu Rev Neurosci. 2015;38:47–68. doi:10.1146/annurev-neuro-071714-034136 25840007

[113] Ahlskog JE. Common Myths and Misconceptions That Sidetrack Parkinson Disease Treatment, to the Detriment of Patients. Mayo Clin Proc. 2020;95(10):2225–2234. doi:10.1016/j.mayocp.2020.02.006 33012351

[114] Salinas MR, Chambers EJ, Ho T, Patient perceptions and knowledge of Parkinson’s disease and treatment (KnowPD). Clin Park Relat Disord. 2020;3:100038. Published 2020 Jan 16. doi:10.1016/j.prdoa.2020.100038 34316624 PMC8298769

[115] Jones HJ, Heron J, Hammerton G, Investigating the genetic architecture of general and specific psychopathology in adolescence. Transl Psychiatry. 2018;8(1):145. Published 2018 Aug 8. doi:10.1038/s41398-018-0204-9 30089819 PMC6082910

[116] Healey ML, Grossman M. Cognitive and Affective Perspective-Taking: Evidence for Shared and Dissociable Anatomical Substrates. Front Neurol. 2018;9:491. Published 2018 Jun 25. doi:10.3389/fneur.2018.00491 29988515 PMC6026651

[117] van Dijk MT, Murphy E, Posner JE, Talati A, Weissman MM. Association of Multigenerational Family History of Depression With Lifetime Depressive and Other Psychiatric Disorders in Children: Results from the Adolescent Brain Cognitive Development (ABCD) Study. JAMA Psychiatry. 2021;78(7):778–787. doi:10.1001/jamapsychiatry.2021.0350 33881474 PMC8060885

[118] Schapira AH, Olanow CW. Neuroprotection in Parkinson disease: mysteries, myths, and misconceptions. JAMA. 2004;291(3):358–364. doi:10.1001/jama.291.3.358 14734599

[119] Niarchou M, Zammit S, Lewis G. The Avon Longitudinal Study of Parents and Children (ALSPAC) birth cohort as a resource for studying psychopathology in childhood and adolescence: a summary of findings for depression and psychosis. Soc Psychiatry Psychiatr Epidemiol. 2015;50(7):1017–1027. doi:10.1007/s00127-015-1072-8 26002411

[120] Spadoni AD, Vinograd M, Cuccurazzu B, Contribution of early-life unpredictability to neuropsychiatric symptom patterns in adulthood. Depress Anxiety. 2022;39(10–11):706–717. doi:10.1002/da.23277 35833573 PMC9881456

[121] de Boer A, Vermeulen K, Egger JIM, *EHMT1* mosaicism in apparently unaffected parents is associated with autism spectrum disorder and neurocognitive dysfunction. Mol Autism. 2018;9:5. Published 2018 Jan 25. doi:10.1186/s13229-018-0193-9 29416845 PMC5784506

[122] Marathe S, Hunter BM, Chakravarthi I, Shukla A, Murray SF. The impacts of corporatisation of healthcare on medical practice and professionals in Maharashtra, India. BMJ Glob Health. 2020;5(2):e002026. Published 2020 Feb 11. doi:10.1136/bmjgh-2019-002026 PMC7042603 32133190

[123] Turner S, Wright JS. The corporatization of healthcare organizations internationally: A scoping review of processes, impacts, and mediators. Public Administration. 2022 Jun;100(2):308–23.

[124] Harding April; Preker Alexander S.. 2000. The Corporatization of Public Hospitals. HNP discussion paper series;. World Bank, Washington, DC. © World Bank. https://openknowledge.worldbank.org/handle/10986/13694 License: CC BY 3.0 IGO

[125] Virk A, Croke K, Mohd Yusoff M, Hybrid Organizations in Health Systems: The Corporatization of Malaysia’s National Heart Institute. Health Syst Reform. 2020;6(1):e1833639. doi:10.1080/23288604.2020.1833639 33314988

[126] Helmchen H Ethische Implikationen der Neurowissenschaften in der Klinik [Ethical aspects of clinical neuroscience]. Nervenarzt. 2000;71(9):700–708. doi:10.1007/s001150050653 11042864

[127] Cotter K, Siskind CE, Sha SJ, Hanson-Kahn AK. Positive Attitudes and Therapeutic Misconception Around Hypothetical Clinical Trial Participation in the Huntington’s Disease Community. J Huntingtons Dis. 2019;8(4):421–430. doi:10.3233/JHD-190382 31594242 PMC6839474

[128] Castillo EG, Ijadi-Maghsoodi R, Shadravan S, Community Interventions to Promote Mental Health and Social Equity. Curr Psychiatry Rep. 2019;21(5):35. Published 2019 Mar 29. doi:10.1007/s11920-019-1017-0 30927093 PMC6440941

[129] Martin LT, Plough A, Carman KG, Leviton L, Bogdan O, Miller CE. Strengthening Integration Of Health Services And Systems. Health Aff (Millwood). 2016;35(11):1976–1981. doi:10.1377/hlthaff.2016.0605 27834236

[130] Álvarez-Díaz JA. Neuroetica como neurociencia de la etica [Neuroethics as the neuroscience of ethics]. Rev Neurol. 2013;57(8):374–382. 24081892

[131] Racine E, Dubljević V, Jox RJ, Can Neuroscience Contribute to Practical Ethics? A Critical Review and Discussion of the Methodological and Translational Challenges of the Neuroscience of Ethics. Bioethics. 2017;31(5):328–337. doi:10.1111/bioe.12357 28503831

[132] Figueroa G Neuroethics: the pursuit of transforming medical ethics in scientific ethics. Biol Res. 2016;49:11. Published 2016 Feb 20. doi:10.1186/s40659-016-0070-y 26897168 PMC4761160

[133] Decety J, Yoder KJ. The Emerging Social Neuroscience of Justice Motivation. Trends Cogn Sci. 2017;21(1):6–14. doi:10.1016/j.tics.2016.10.008 27865787

[134] Sobański JA, Dudek D. Psychiatry and neurology: from dualism to integration. Neurol Neurochir Pol. 2013;47(6):577–583. doi:10.5114/ninp.2013.39069 24375004

[135] Taylor JJ, Williams NR, George MS. Beyond neural cubism: promoting a multidimensional view of brain disorders by enhancing the integration of neurology and psychiatry in education. Acad Med. 2015;90(5):581–586. doi:10.1097/ACM.0000000000000530 25340364 PMC4405399

[136] Sachdev P, Mohan A. An International Curriculum for Neuropsychiatry and Behavioural Neurology. Rev Colomb Psiquiatr. 2017;46 Suppl 1:18–27. doi:10.1016/j.rcp.2017.05.001 29037334

[137] Trapp NT, Martyna MR, Siddiqi SH, Bajestan SN. The Neuropsychiatric Approach to the Assessment of Patients in Neurology. Semin Neurol. 2022;42(2):88–106. doi:10.1055/s-0042-1745741 35477181 PMC9177704

[138] Schildkrout B, Benjamin S, Lauterbach MD. Integrating Neuroscience Knowledge and Neuropsychiatric Skills Into Psychiatry: The Way Forward. Acad Med. 2016;91(5):650–656. doi:10.1097/ACM.0000000000001003 26630604

[139] Benjamin S, Widge A, Shaw K Neuropsychiatry and neuroscience milestones for general psychiatry trainees. Acad Psychiatry. 2014;38(3):275–282. doi:10.1007/s40596-014-0112-0 24715675

[140] Coverdale J, Balon R, Beresin EV, Teaching clinical neuroscience to psychiatry residents: model curricula. Acad Psychiatry. 2014;38(2):111–115. doi:10.1007/s40596-014-0045-7 24493360

[141] Gopalan P, Azzam PN, Travis MJ, Schlesinger A, Lewis DA. Longitudinal interdisciplinary neuroscience curriculum. Acad Psychiatry. 2014;38(2):163–167. doi:10.1007/s40596-014-0049-3 24519799

[142] Cooper JJ, Korb AS, Akil M. Bringing Neuroscience to the Bedside. Focus (Am Psychiatr Publ). 2019;17(1):2–7. doi:10.1176/appi.focus.20180033 31975952 PMC6493145

[143] Sachdev PS, Mohan A. Neuropsychiatry: where are we and where do we go from here?. Mens Sana Monogr. 2013;11(1):4–15. doi:10.4103/0973-1229.109282 23678234 PMC3653233

[144] Fogel BS, Schiffer RB. Defining neuropsychiatry: professional activities and opinions of psychiatrist-neurologists with dual certification. J Neuropsychiatry Clin Neurosci. 1989;1(2):173–175. doi:10.1176/jnp.1.2.173 2521059

[145] Ibáñez A, García AM, Esteves S, Social neuroscience: undoing the schism between neurology and psychiatry. Soc Neurosci. 2018;13(1):1–39. doi:10.1080/17470919.2016.1245214 27707008 PMC11177280

[146] Perez DL, Keshavan MS, Scharf JM, Boes AD, Price BH. Bridging the Great Divide: What Can Neurology Learn From Psychiatry?. J Neuropsychiatry Clin Neurosci. 2018;30(4):271–278. doi:10.1176/appi.neuropsych.17100200 29939105 PMC6309772

[147] Lee TS, Ng BY, Lee WL. Neuropsychiatry--an emerging field. Ann Acad Med Singap. 2008;37(7):601–605. 18695776

[148] Fricchione GL. Evolving a new neuropsychiatry. Dialogues Clin Neurosci. 2018;20(2):141–145. doi:10.31887/DCNS.2018.20.2/gfricchione 30250391 PMC6136125

[149] Van Oudenhove L, Cuypers SE. The philosophical “mind-body problem” and its relevance for the relationship between psychiatry and the neurosciences. Perspect Biol Med. 2010;53(4):545–557. doi:10.1353/pbm.2010.0012 21037408

[150] Frisch S Are Mental Disorders Brain Diseases, and What Does This Mean? A Clinical-Neuropsychological Perspective. Psychopathology. 2016;49(3):135–142. doi:10.1159/000447359 27428178

[151] Frisch S Why Biological Psychiatry Hasn’t Delivered Yet - and Why Neurology Knows. Psychiatry Investig. 2021;18(12):1145–1148. doi:10.30773/pi.2021.0258 PMC8721299 34872239

[152] Martin JB. The integration of neurology, psychiatry, and neuroscience in the 21st century. Am J Psychiatry. 2002;159(5):695–704. doi:10.1176/appi.ajp.159.5.695 11986119

[153] Northoff G. Neuropsychiatry. An old discipline in a new gestalt bridging biological psychiatry, neuropsychology, and cognitive neurology. Eur Arch Psychiatry Clin Neurosci. 2008;258(4):226–238. doi:10.1007/s00406-007-0783-6 18297424

[154] Peled A Plasticity imbalance in mental disorders the neuroscience of psychiatry: implications for diagnosis and research. Med Hypotheses. 2005;65(5):947–952. doi:10.1016/j.mehy.2005.05.007 15996829

[155] Kaushik A, Kostaki E, Kyriakopoulos M. The stigma of mental illness in children and adolescents: A systematic review. Psychiatry Res. 2016;243:469–494. doi:10.1016/j.psychres.2016.04.042 27517643

[156] Oexle N, Waldmann T, Staiger T, Xu Z, Rüsch N. Mental illness stigma and suicidality: the role of public and individual stigma. Epidemiol Psychiatr Sci. 2018;27(2):169–175. doi:10.1017/S2045796016000949 27919303 PMC6998948

[157] Avdibegović E, Hasanović M. The Stigma of Mental Illness and Recovery. Psychiatr Danub. 2017;29(Suppl 5):900–905. 29283987

[158] Corrigan PW, Rao D. On the self-stigma of mental illness: stages, disclosure, and strategies for change. Can J Psychiatry. 2012;57(8):464–469. doi:10.1177/070674371205700804 22854028 PMC3610943

[159] Abdullah T, Brown TL. Mental illness stigma and ethnocultural beliefs, values, and norms: an integrative review. Clin Psychol Rev. 2011;31(6):934–948. doi:10.1016/j.cpr.2011.05.003 21683671

[160] Bravo-Mehmedbašić A, Kučukalić S. Stigma of psychiatric diseases and psychiatry. Psychiatr Danub. 2017;29(Suppl 5):877–879. 29283982

[161] Tyerman J, Patovirta AL, Celestini A. How Stigma and Discrimination Influences Nursing Care of Persons Diagnosed with Mental Illness: A Systematic Review. Issues Ment Health Nurs. 2021;42(2):153–163. doi:10.1080/01612840.2020.1789788 32762576

[162] Tedrus GMAS Pereira RB, Zoppi M, Epilepsy, stigma, and family. Epilepsy Behav. 2018;78:265–268. doi:10.1016/j.yebeh.2017.08.007 29126703

[163] Stangl AL, Earnshaw VA, Logie CH, The Health Stigma and Discrimination Framework: a global, crosscutting framework to inform research, intervention development, and policy on health-related stigmas. BMC Med. 2019;17(1):31. Published 2019 Feb 15. doi:10.1186/s12916-019-1271-3 30764826 PMC6376797

